# Antitumor Activity of Curcumin in Glioblastoma

**DOI:** 10.3390/ijms21249435

**Published:** 2020-12-11

**Authors:** Blake C. Walker, Sandeep Mittal

**Affiliations:** Carilion Clinic Neurosurgery, Fralin Biomedical Research Institute at VTC, Virginia Tech Carilion School of Medicine, Roanoke, VA 24016, USA; bcwalker@carilionclinic.org

**Keywords:** curcumin, turmeric, malignant gliomas, glioblastoma, anticancer drug, nutraceutical

## Abstract

Current standard-of-care treatment for glioblastoma, the most common malignant primary central nervous system (CNS) tumor, consists of surgical resection followed by adjuvant chemotherapy and radiation (Stupp protocol), providing an overall median survival of 15 months. With additional treatment using tumor-treating fields (Optune^®^ therapy, Novocure Ltd., Haifa, Israel), survival can be extended up to 20 months. In spite of significant progress in our understanding of the molecular pathogenesis, the prognosis for patients with malignant gliomas remains poor and additional treatment modalities are critically needed. Curcumin is a bright yellow pigment found in the rhizome of the widely utilized spice, turmeric (*Curcuma longa*). It has long been used in South Asian traditional medicines and has been demonstrated to have in vitro antioxidant, anti-inflammatory, and antiproliferative effects. Curcumin has been demonstrated to induce multiple cytotoxic effects in tumor cells including cell cycle arrest, apoptosis, autophagy, changes in gene expression, and disruption of molecular signaling. Additionally, curcumin has been shown to potentiate the effect of radiation on cancer cells, while exhibiting a protective effect on normal tissue. Curcumin’s positive safety profile and widespread availability make it a promising compound for future clinical trials for high-grade gliomas.

## 1. Introduction

Primary central nervous system (CNS) tumors represent a heterogeneous group of pathologies with variable prognosis, treatment, life expectancy, and molecular profiles. Glioblastoma (GBM) is the most common primary malignant CNS tumor, with an estimated incidence of 3.21 per 100,000 people per year. Additionally, GBM accounts for 57.7% of all gliomas and 48.6% of all malignant brain tumors based on the most recent Central Brain Tumor Registry of the United States (CBTRUS) data collected from 2013–2017 [1]. Despite considerable improvement in our understanding of the underpinnings of the disease over the past two decades, the overall prognosis for GBM remains dismal. Historically, treatment consisted of maximal surgical resection with radiotherapy alone and had a median overall survival time of approximately 12.1 months. The addition of adjuvant temozolomide (TMZ), an alkylating agent, to maximal surgical resection and radiotherapy (Stupp protocol) had a modest increase in median overall survival time to 14.6 months [2]. When low-intensity alternating tumor-treating electrical fields (Optune^®^ therapy, Novocure Ltd., Haifa, Israel) are combined to maximal surgical resection followed by the Stupp regimen, median overall survival time is prolonged to 20.9 months [3]. Unfortunately, additional chemotherapeutic agents are frequently limited by side effect profiles. As such, compounds with low toxicity are frequently being examined to augment the effects of current radiation and chemotherapy regimens. In this review, we aim to provide an updated review of the literature on the effects of curcumin in malignant gliomas particularly focusing on experimental evidence from the last 5 years.

**Figure 1:**
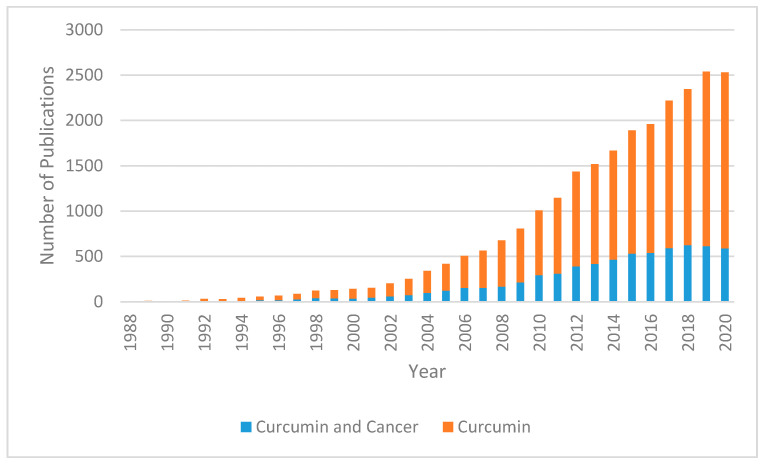
Number of publications since 1988 (sourced from PubMed Database). The blue portion of the bar represents proportion of results returned with search terms “curcumin” and “cancer” while the orange portion represents the total number of studies returned with the search term “curcumin”.

Curcumin (diferuloymethane) is a yellow pigment derived from the rhizome of turmeric (*Curcuma longa*) and has been used in traditional Indian Ayurvedic and Southeast Asian medicine for millennia; it has traditionally been known to have anti-inflammatory effects [4]. Demethoxycurcumin and bis-demethoxycurcumin are the main metabolites of curcumin and belong to a class of chemicals called diarylheptanoids [5]. In recent years, the multiple molecular pathways that curcumin modulates have been studied extensively. Downstream effects on enzymes, including kinases, growth factors, receptors, transcriptional factors, and inflammatory cytokines may support evidence of its antitumor effects [6]. Curcumin has been shown to decrease malignant characteristics of GBM stem cells, potentiate the effects of modern chemotherapy and radiation while protecting normal tissue, and selectively induce apoptosis in cancer cells [7,8,9,10]. In recent years, the number of curcumin-related publications has been trending upward (Figure 1).

## 2. Chemical Structure and Properties of Curcumin

**Figure 2:**
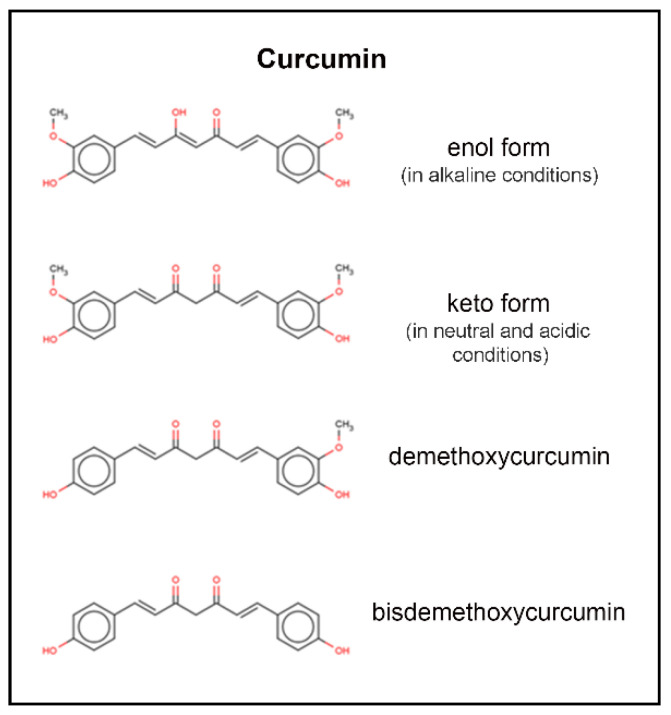
Molecular structure of curcumin and its tautomers. From top to bottom; curcumin in its enol form, curcumin in its keto form, demethoxycurcumin, and bisdemethoxycurcumin.

The molecular formula of curcumin [1,7-bis-(4-hydroxy-3-methoxyphenyl)-1,6-heptadiene-3,5-dione], is C_21_H_22_O_6_. Curcumin has a molecular weight of 368.38 daltons. Keto-enol tautomerism of curcumin depends on the acidity of the solution, with enol form predominating in alkaline media and keto form predominating in acidic and neutral media (Figure 2) [5]. At acidic to neutral pH, curcumin solutions are yellow, while alkaline solutions are orange to red in color. Degradation has been shown to follow second-order kinetics in various buffer systems from pH 1–11, as well as in fixed ionic systems [5]. Curcumin is insoluble in water at room temperature and neutral pH (log P between 2.3 and 3.2), and degrades rapidly at neutral and alkaline pH; this is complicated by the fact that curcumin is more stable at acidic pH, with an equilibrium shift toward the less stable, neutral form [11]. Degradation of curcumin outside of biological systems occurs via solvolysis and oxidative degradation, with photodegradation also contributing to a five percent loss of parent compound when prepared in clear versus amber glass [11].

## 3. Pharmacokinetics of Curcumin

Curcumin is the most biologically active component of turmeric, comprising 2–8% of preparation volume. Systemic bioavailability is low secondary to first-pass metabolism. Intestinal metabolism has also been implicated in poor bioavailability to curcumin via glucuronidation and sulfation. Doses at approximately 3.6 g can be detected in colorectal tissue [12]. This, however, appears to be a local effect as transport across the intestinal mucosa is very poor and molecules that do enter the blood stream are subject to rapid chemical modification in the blood, liver, and other organs. A single oral dose of 500–8000 mg of curcumin could not be detected in serum levels of human subjects from one to four hours after administration [13]. The in vitro and in vivo stabilities of curcumin are very poor, with a half-life of five minutes [11]. Excretion occurs via the biliary, and to a much lesser extent, renal systems [13]. Given the poor systemic bioavailability of curcumin, in vivo studies have trailed in vitro studies in terms of generation of anticancer effects, outside of GI malignancy. It is postulated that plasma concentrations of curcumin after oral administration are so low relative to the LC50 that curcumin in its oral preparation would not have tumoricidal effects outside of GI neoplasms [13]. It is, however, important to note that the conjugated metabolites, curcumin glucuronide and curcumin sulfite, retain most of the structure of curcumin. These conjugated curcumin compounds have recently been shown to have antitumorigenic and anti-inflammatory effects and likely would not measure in plasma concentrations of curcumin after oral dosing [14]. Additionally, there is limited evidence to suggest that curcumin crosses the blood–brain barrier in humans; however, its exact mechanism is not well described.

## 4. Methods of Increasing Bioavailability

Several factors affecting bioavailability of curcumin have been identified including its large crystalline structure, low water solubility, and extensive metabolism. Curcumin preparations and delivery vehicles that address curcumin’s low bioavailability have yielded large increases in bioavailability. Studies involving synthetic curcumin analogs with greater bioavailability, such as difluorinated-curcumin, have also been shown to have antitumor effects [14].

One method of overcoming low bioavailability is to decrease the size of the bioactive agent, therefore increasing its solubility; this has been the focus of improving the preparations of curcumin over the past years [15]. A limitation to producing ingestible medications is the solvents in which they are prepared with. Low-crystallinity nanoparticles are the most recent “green” solutions to the bioavailability issues of curcumin. Traditionally, low-crystallinity nanoparticles were generated by exposure to various toxic solvents, such as chloroform, or via the formation of cyclodextrin complexes, which would limit medical application. A novel method of generating low-crystallinity curcumin nanoparticles was formulated by integrating low-crystallinity curcumin into a nanoporous starch aerogel (NSA). After incorporation into the NSA, the curcumin-NSA matrix was subjected to drying by exposure to supercritical carbon dioxide. Through precipitation by pressure reduction of gas-expanded liquid (PPRGEL), spherical curcumin nanoparticles can subsequently form due to hydrophobic effect, with particle size averaging from 66 to 71 nM depending on temperature. At room temperature curcumin NSA nanoparticles increased the bioavailability of curcumin by 173-fold (0.4% curcumin vs. 69.1% low-crystallinity curcumin nanoparticles) [15]. Poly-lactic-co-glycolic acid (PLGA) nanoformulated curcumin has been shown to have three to four times greater concentration and longevity in CNS tissue at similar oral doses when compared to standard curcumin preparations in a murine model [16]. Nanosuspension preparations of curcumin increase saturation solubility and dissolution velocity by the creation of pure drug crystals with accompanying stabilizer, with more modest increases in bioavailability by 4.2-fold [17]. It is important to note that high drug concentration can be achieved with a relatively low number of stabilizers and this preparation has been extensively studied via multiple routes of administration including oral, intramuscular, intravenous, ocular and pulmonary systems [18].

Liposomes have been used as a drug delivery system for decades. Liposomal curcumin can be prepared by film dispersion-pH gradient, utilizing egg phosphatidylcholine, polyethylene glycol, and 1,2,-distearoylphosphatidylethanolamine, and 1,2-distearoyl-*sn*-glycero-3-phosphoethanolamine-N-[amino(polyethylene glycol)-2000]. Utilizing a rotary vacuum evaporator, a lipid film can be generated and hydrated [19]. Liposomal curcumin increases the bioavailability of curcumin. Affixing a transport molecule to the liposomal curcumin, such as p-Aminophenyl-α-D-mannopyranoside (MAN), enhances transport across the blood–brain barrier. Furthermore, liposomes modified with MAN appear to preferentially target the cortex, cerebellum, brainstem, hippocampus, and pontine nuclei [19]. Curcumin micelles are another form of increased bioavailability curcumin, approximately 19-fold greater than standard preparation curcumin [20]. Curcumin micelles can be formed by disodium glycyrrhizin with an amorphous solid dispersion of curcumin, prepared by mechanism of mechanical ball milling and have been shown to have increased cell permeability and cytotoxic effects in rat models [20]. One drawback of micelle preparations is that the mass of stabilizers and excipients (i.e., “inactive ingredients”) required for such preparations may have their own undesired side effects, including hemolysis; this may render micelles a less attractive method of drug delivery [18]. Methods of increasing bioavailability as well as enhancing delivery to specific cellular targets will become increasingly important with translational studies.

## 5. Glioma-Related Pharmacodynamics of Curcumin

### 5.1. Curcumin Inhibits Matrix Metalloproteinase Expression

Matrix metalloproteinases (MMPs) have been implicated in tumor invasiveness. MMPs are a category of zinc-dependent proteolytic endopeptidases that allow tumor cells to become locally invasive by degrading extracellular matrix proteins, growth factor binding protein, and cell adhesion molecules [21]. MMPs have been shown to cause disseminated tumor growth. MMP-2 has been shown to be required for angiogenesis and vasculogenesis via vascular endothelial growth factor (VEGF) A expression and coexpression with VEGF receptor 2 (VEGFR2) [21]. MMP-9 has been shown to be required for vasculogenesis alone, exerting its effect independent of the VEGF pathway via recruitment of endothelial and myeloid precursors [22]. Smaller diameter gliomas have been shown to have lower expression of MMP-2, whereas MMP-2 is highly expressed in larger diameter malignant gliomas, with a corresponding decrease in expression of a group of inhibitory proteins, tissue inhibitors of metalloproteinases (TIMPs) [21]. U373 human GBM cells treated with variable concentrations of curcumin in DMSO were shown to have reduced expression of MMP-2, 9, 14, 15, 16, 17, 24, and 25, compared to untreated cells [23]. Cell invasion assays performed with SNB19 and A1027 human GBM cells resulted in significantly less cellular migration at 10, 15, and 20 μM concentrations compared to controls [9].

### 5.2. Curcumin Downregulates the STAT3 Pathway

Survivin, or baculoviral inhibitor of apoptosis (BIRC5), is a protein belonging to the inhibitor of apoptosis (IAP) family of proteins; it is expressed in embryonal tissues, upregulated in human cancer cells and absent in most normal tissue [24]. Survivin has multiple functions including cell cycle regulation as well as inhibition of apoptosis and has been shown to induce chromosomal instability [25]. In a recent study, 144 patients with GBM underwent whole-genome sequencing and prognosis of the group with high survivin expression was found to be poor, compared to those with lower expression [25]. Patient-derived GBM cells and established human GBM cell lines (U87, U51, U235) treated with 25 μM curcumin have been shown to have increased expression of phosphorylated ERK, p38 and JNK (via c-Jun) at one and six hours. Phosphorylated ERK represses STAT3 through phosphorylation at Ser727 and dephosphorylation at Tyr705, ultimately resulting in inactivation of STAT3 and inability to translocate into the nucleus causing decreased expression of survivin and IAP2 [8]. U373 human GBM cells treated with escalating variables of curcumin in DMSO and analyzed via flow cytometry were shown to selectively induce apoptosis at lower concentrations (10 μg/mL) with similar levels of apoptosis and necrosis at higher concentrations (20 μg/mL) [23].

### 5.3. Curcumin Inhibits Wnt and HDGF Pathways

The Wnt signaling pathway is an important part of adult tissue homeostasis and can induce mitogenic stimulation, cell fate specification, and differentiation via a signal cascade that begins on the surface of signal cells that bind to the Frizzled/low-density lipoprotein receptor-related protein complex at the target cell, resulting in signal transduction via Dishevelled, glycogen synthase kinase-3β, Axin, and adenomatous polyposis coli (APC), ultimately resulting in increased intracytoplasmic and intranuclear β-catenin concentration [26]. Wnt signaling pathway regulation has been shown to be an important regulator of early brain development and is essential for presynaptic and postsynaptic transcriptional regulation, with dysregulation of Wnt having been associated with development of tumors, including GBM [27]. Hepatoma-derived growth factor (HDGF) is an angiogenesis-promoting growth factor that is upregulated in gliomas, which ultimately forms a complex with β-catenin from the Wnt pathway; this upregulation from Wnt and HDGF can promote tumor generation, progression and metastasis [28]. U251 and LN229 human brain GBM cell lines cultured in curcumin concentrations ranging from 5 to 200 μmol/L were found to have decreased proliferation, invasion, and migration distance. Furthermore, curcumin was shown to inhibit HDGF, and consequently the complex formed between HDGF and β-catenin (which has been shown to promote tumorigenesis) was reduced [28].

### 5.4. Curcumin Modulates Protein Ubiquitination

Protein ubiquitination is an evolutionary conserved cascade that is important for posttranslational genomic reorganization. The dysfunction of E3 ubiquitin ligase, a component of the ubiquitin pathway, has been shown to be involved in a number of malignancies [29]. Neural precursor cell-expressed developmentally downregulated protein 4 (NEDD4) is an E3 ubiquitin ligase responsible for substrate recognition in the ubiquitin pathway, ultimately leading to protein degradation [9]. NEDD4 has an oncogenic effect by facilitating PTEN degradation via ubiquitination; the downstream effect is activation of the PI3K/AKT pathway and cellular proliferation [30]. Western blot and real-time PCR (qPCR) analysis of SNB190 and A1027 human GBM cells treated in 15 μM curcumin for 72 h revealed decreased expression of NEDD4. Cellular lines with NEDD4 siRNA knockdowns alone exhibited decreased proliferation compared to control, while cells treated with a combination of NEDD4 siRNA knockdown and 15 μM curcumin exhibited significantly less proliferation compared to control and knockdown alone [9].

S-phase kinase protein 2 (Skp2), is a component of the Skp2-SCF E3 ligase complex, involved in conjugation of K48-linked ubiquitin chains to induce proteasome-mediated proteolysis, and conjugation of K63-linked ubiquitin chains to modulate substrate function [31]. Skp2 also forms the Skp-Cullin-F box complex, which is responsible for ubiquitin-mediated degradation of the G1 checkpoint CDK inhibitors, G21, P21 and P27, resulting in cell cycle progression [32,33]. Skp2 overexpression has been implicated in tumorigenesis and has been associated with multiple human cancers, including gliomas [31,34,35]. Knockdown of Skp2 has been shown to increase sensitivity of gliomas to TMZ, attenuate growth of glioma cells and induce senescence in glioma cells [33]. U251 and SNB19 human GBM cells treated with variable curcumin preparations were found to have decreased Skp2 expression via RT-PCR, as well as decreased migration, invasion, and proliferation; viral transfection with Skp2 cDNA rescued growth inhibition by curcumin [36].

### 5.5. Curcumin Upregulates RANK

The receptor activator of NF-κB (RANK), or tumor necrosis factor (TNF) receptor superfamily member 11A, via interaction with TNF-receptor-associated factors, induces transduction of stimulation signals to NF-κB and c-Jun N-terminal kinase [37]. RANK/RANKL signaling has been shown to upregulate survival proteins through Akt and EGFR pathways, or promote apoptosis similar to other TNFs [37]. As previously discussed, STAT3 inhibition causes decreased expression of survival proteins [8]. RANK expression is negatively modulated by STAT3 and inhibition of STAT3 via knockdown, or exposure to curcumin preparation has been shown to increase RANK expression in U87 and U251 GBM cells [37]. STAT3 may very well be an attractive target for treatment of CNS tumors, and curcumin has been shown to inhibit this pathway through its interaction with ERK [8].

### 5.6. Curcumin Promotes Apoptosis

Carbobenzoxy-Leu-Leu-leucinal (MG132) is a proteasome inhibitor involved in the degradation of connexin 43, a gap junction protein associated with enhanced glioma invasiveness [38]. Connexin 43 (Cx43) overexpression has been found in TMZ-resistant cells [39]. Exposure of TMZ-resistant GBM cells to 10 μM curcumin increased apoptosis two-fold (from 4% to 8%), with a resulting decrease in Cx43 expression by 40%; subsequent exposure to MG132 negated Cx43 degradation, implicating the ubiquitin–proteasome pathway in Cx43 degradation [39]. This demonstrates yet another downstream effect of the ubiquitin pathway that curcumin interacts with.

### 5.7. Curcumin Induces Oxidative Stress in Glioma Cell Lines

Direct cytotoxic effects of curcumin in cancer cells have also been observed via production of reactive oxygen species (ROS). Patient-derived and immortalized GBM cells (U87, U51, U235) treated with variable curcumin concentrations reached viability levels less than 20% at concentrations of 70 μM at 72 h; the effect appeared to be dose-dependent. Immunofluorescence using a general oxidative stress indicator molecular probe (CM-H2DCFDA) demonstrated induction of ROS at one and six hours with concentrations of 25 μM, returning to control levels after 24 h, and appears to be counteracted by pretreating with *N*-acetylcysteine, an antioxidant [8]. Radiation therapy partially exerts its effects via generation of ROS. Curcumin preparations have been shown to cause radiosensitization of neoplastic tissue, likely through interactions with previously discussed apoptotic pathways, while protecting normal tissue via reduction in oxidative stress and inflammatory response [40].

### 5.8. Curcumin and NRF2

The nuclear factor-erythroid 2 related factor 2 (NRF2) is a regulator of oxidative stress response. NRF2 is usually bound to KEAP1, until cellular exposure to chemical toxins and radiation causes its disassociation and intranuclear accumulation resulting in activation of genes related to antioxidant response and detoxification, ultimately increasing resistance to oxidative stress [41]. NRF2 has been postulated to increase GBM survival, especially given the elevated oxidative stress in the GBM microenvironment secondary to peroxisome activity, glucose metabolism, mitochondrial dysfunction, and oncogene activity [41]. NRF2 has also been associated with reduced ferroptosis in GBM [42]. Knockdown of NRF2 in U87MG GBM cell lines that were subsequently treated with radiation resulted in downregulation of oxidative stress proteins as well as decreased proliferative and self-renewal capacity compared to control groups that did not have an NRF2 knockdown [41]. Patients with GBM and upregulated NRF2 have reduced overall survival compared to patients with normal NRF2 levels [42]. Curcumin has been shown to induce protective mechanisms against oxidative stress and is a known activator of NRF2 [43]. Although NRF2 activation from curcumin exposure in GBM is possible, it is unlikely that, in this context, it would result in an overall increase in survival. NRF2 has also been postulated to have a neuroprotective effect and its upregulation may be beneficial in the treatment of neurodegenerative diseases [44,45]. The net effect of curcumin exposure in GBM cell lines in vitro is decreased malignant characteristics, potentiation of chemotherapy effect and increased apoptosis in cancer cells [7,8,9,10].

**Figure 3:**
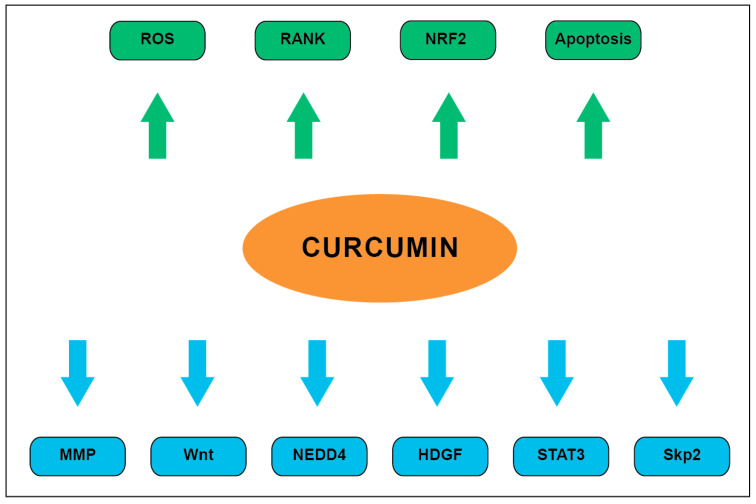
Summary of well described curcumin pharmacodynamics. Downregulated processes are denoted by blue arrows while upregulated processes are denoted by green arrows.

Overall, curcumin has been shown to exert numerous antitumor effects on various molecular pathways and cellular processes as summarized in Figure 3.

## 6. Toxicity

Allowable daily intake of curcumin can reach 3 mg/kg/day and the toxicity profile is favorable; people taking 500–12,000 mg doses reported headache, rash, and yellow stool [46]. It is important to note, however, that this dose range will likely not produce a serum concentration high enough to potentially treat CNS pathology. Due to its broad activity against a number of enzymes, curcumin and its degradation products may exert potential toxicity via interaction with hERG channels and inhibition of cytochrome P450 and glutathione S-transferase, producing cardiotoxicity and drug–drug interactions, respectively; these are the main potential side effects, which have been largely studied in rat models [11]. Despite its widely demonstrated anti-cancer effects, curcumin has been reported to have cytotoxic effects on normal human lymphocytes, as well as on human kidney cells and murine macrophage cell lines at IC50 concentrations of 15.2 and 31 μM, respectively [11]. It is also important to note that the US Food and Drug Administration has classified curcumin as “Generally Recognized as Safe”, or GRAS as of November 2018. This designation is for intended use, which at the moment includes use as an ingredient in various food categories, at addition levels of 0.5 to 100 mg/100 g and does not include use as a supplement or treatment for any condition.

## 7. Other Considerations

### 7.1. PAINS

Curcumin has been designated as a pan-assay interference compounds (PAINS) and has been shown to have activity in multiple types of assays via interference with assay results, rather than having true interactions with targets. Curcumin exhibits all of the PAINS compound behaviors, including covalent labeling of proteins, structural decomposition, membrane disruption, fluorescence interference, metal chelation, redox reactivity, and aggregation [11]. Care must be taken when reviewing the literature, as well as when designing studies to ensure that appropriate assays are selected to avoid erroneous results, specifically, generating research based on scientific questions rather than pan-assays.

### 7.2. IMPs

Invalid metabolic panaceas (IMPs) are compounds that have multiple reported bioactive properties and a high ratio of positive activities witnessed in proportion to total reported activities. IMPs compounds are characterized by many of the PAINS criteria listed above, in addition to a reactive Michael acceptor (part of an organic chemistry reaction) and fluorescence activity. Curcumin’s 28 reported pharmacological activities (less than 10% of total observed reactions) in the NAPRALERT database account for 50% of the total reported activities [11]. A favorable percentage, observed with other drugs such as paclitaxel, ranges between one and two percent. Additionally, the high degree to which curcumin reacts across many bioassays places it among the top 100 most bioactive compounds [11].

## 8. Conclusions

Curcumin is an attractive natural product that has a multitude of proposed health benefits. Its safety profile secondary to low toxicity has been time-tested and the product has been a component of human diet for thousands of years. As more data emerge regarding its interaction with Wnt, RANK, STAT3, and other signal cascades or tumorigenic molecules, it is becoming clear that curcumin has the potential to have in vivo anti-glioma effects. Care must be taken to ensure that study design accounts for curcumin’s promiscuous bioactivity profile, and to control for potential false positive results from intrinsic fluorescence. Other hurdles to overcome include improving CNS drug delivery, where progress is actively being made with novel drug delivery systems. Translational studies involving human glioma cell lines and curcumin are essential to advance understanding and to generate data based on questions and the scientific method, rather than pan-assays. At this moment, there is a good deal of high-quality in vitro data to suggest that curcumin has the potential to complement current standard of care chemotherapy and radiation.
